# Phenotype-endotype relationship in elderly atopic dermatitis and effects of dupilumab therapy: prospective study

**DOI:** 10.1007/s00403-025-04090-5

**Published:** 2025-03-17

**Authors:** Giuseppina Caiazzo, Maddalena Napolitano, Maria Quaranta, Vincenzo Picone, Gabriella Fabbrocini, Cataldo Patruno

**Affiliations:** 1 Department of Advanced Biomedical Sciences, University of Naples Federico II, Napoli, Italy; 2 Section of Dermatology, Department of Clinical Medicine and Surgery, University of Naples Federico II, Naples, Italy; 3 Department of Medicine and Health Sciences Vincenzo Tiberio, University of Molise, Campobasso, Italy

**Keywords:** Elderly, Atopic dermatitis, Dupilumab, Endotype, Phenotype

## Abstract

Atopic dermatitis is a chronic inflammatory systemic disease that can persist or start in adulthood, even in elderly age, with several clinical phenotypes. The aim of this study was to evaluate the relationship between phenotype and endotype in elderly patients, and the long-term effects of dupilumab on molecular signature in these patients. A total of 50 elderly patients had been treated with dupilumab during the follow up period. Regarding effectiveness parameters, mean EASI score at Week 0 was 25.12 ± 4.23 and significantly reduced to 2.42 ± 3.15 at Week 52 (*p* < 0.05). Regarding safety, none of the registered adverse events led to the discontinuation of dupilumab therapy. Moreover, after 52 weeks of dupilumab treatment, Th2 cytokines expression was decreased, with IL-13 and IL-31 downregulated in both patient groups at Week 52, at both gene and protein levels when compared with Week 0. Our data also revealed a significant increase in both IL-4 gene expression and protein production at Week 52 when compared with Week 0. Unexpectedly, our results also revealed that IL-22 cutaneous expression was significantly increased, while circulating levels were decreased at Week 52 when compared to Week 0. In conclusion, our results highlight the effectiveness of dupilumab at a late time point as Week 52. Of note, a dumping of the Th2- and Th17- immune response at both systemic and in situ level and a possible remodelling role of IL-22 in the skin is suggested.

## Introduction

Atopic dermatitis (AD) is a chronic inflammatory systemic disease with a chronic relapsing course that often occurs in childhood, but it can persist or start in adulthood, even in elderly age [1]. The pathogenesis is the result of the complex interplay between genetic underpinnings, altered skin barrier function, skin microbiome changes, and dysregulation of immune system [2, 3]. In this scenario, the overexpression mainly of T helper 2 (Th2) interleukins (IL), such as IL-4, IL-13, IL-31 and other cytokines involved in Th17 and Th22 pathways, such as IL-22, and IL-17 A, have a role in the inflammatory response and also in skin barrier dysfunction by inducing itch and inhibiting the expression of epidermal proteins, such as filaggrin (FLG), claudin-1 (CLD) and involucrine (INV) [3–5]. Aging is associated with skin microbiome alterations and reduced skin barrier function, including decreased barrier repair and downregulation of the structural proteins also involved in AD pathogenesis (FLG, CLD, INV) [6]. Moreover, innate and adaptive immunity changes in older individual (so called inflammaging) have some overlap with hallmarks observed in AD [7]. With aging, pattern recognition receptors decline in function, phagocytic activity of polymorphonuclear leukocytes decreases and increased quantities of IgE-dendritic and mast cells are found in skin [7]. Furthermore, several studies on microbiota across different age groups noted decreased diversity in the microbiota in elderly subjects [7–8]. A high prevalence of Staphylococcus aureus colonization has been noted in the elderly population [8]. Therefore, it is assumed that several factors make the elderly susceptible to AD [6–8]. These features may be associated to some clinical AD patterns more frequently observed with ageing, namely nummular eczema, prurigo nodularis, head-and neck dermatitis, chronic hand eczema, generalized eczema, and erythroderma; on the other hand, classical flexural AD is found in about 10% of cases [9, 10].

Dupilumab is a monoclonal antibody directed against the α-subunit of the receptor shared by IL-4 and IL-13 and used for the treatment of moderate-to-severe AD [11]. It has been demonstrated a good long-term efficacy and safety profile in atopic patients of all ages, even in the elderly patient [10–13]. The aim of this study was to evaluate the relationship between phenotype and endotype in elderly AD patients, and the long-term effects of dupilumab on molecular signature in these patients.

## Materials and methods

A monocentric prospective study was performed including 50 elderly patients (≥ 65 years old) affected by moderate-to-severe AD undergoing treatment with dupilumab and attending the outpatient clinic of the Dermatology Unit, University Federico II of Naples, from March 2022 to April 2023. Dupilumab was administered at labelled dosage (600 mg on day 1, followed by 300 mg every 2 weeks) in all the patients. The study design conformed to the principles of Helsinki Declaration, was approved by the Ethics Committee of our Institution and a written informed consent was given by every subject involved in the study. Each patient met the following inclusion criteria: (I) diagnosis of moderate-to-severe [Eczema Area and Severity Score (EASI) ≥ 24)] AD performed by expert dermatologists and based on clinical presentation, personal history, and Hanifin and Rajka criteria; (II) patients of both sexes, every ethnicity, and ≥ 65 years old. For each patient, the following demographic and clinical data were recorded: age, sex, clinical phenotype of AD [flexural (F) or non-flexural (NF)] ), comorbidities (atopic and non-atopic), adverse events (AEs). According to the Italian Medicines Agency (AIFA) Access Program, all the patients received dupilumab after inefficacy, loss of efficacy, or contraindication of cyclosporine. Patients with 2 or more clinical phenotypes simultaneously were excluded from the study. Efficacy of dupilumab was measured evaluating the following scores: EASI, Pruritus–Numerical Rating Scale (P-NRS), and Dermatology Life Quality Index (DLQI). Evaluations were at baseline (W0), week (W)16, and W52 of therapy. In addition, at W0 and W52, 2 biopsies [1from lesional (LES) skin and 1 from non-lesional (NLES)skin] and a peripheral blood sample were taken from each patient.

### RNA extraction and real-time

RNA was extracted (RNeasy Mini Protocol Qiagen, Valencia, CA) from lesional and non-lesional skin biopsies, according to the manufacturer’s instructions. The RNA yield was determined by quantifying the samples on a Nanodrop ND1000 UV-vis Spectrophotometer. cDNA was prepared using the Transcriptor High fidelity cDNA Synthesis Kit (Roche, Indianapolis, IN, USA). Quantitative reverse transcriptase polymerase chain reaction (qRT-PCR; LightCycler, Roche, Indianapolis, IN, USA) was performed to assess gene expression of main cytokines involved in AD pathogenesis, namely IL-13, IL-4, IL-31, IL-22, IL-17 A, and periostin, by Sybr green assay. PCR primers were designed based on published sequences, and their specificity was verified with BLAST alignment search. The amount of mRNA for a given gene in each sample was normalized to the amount of mRNA of 18 S reference gene in the same sample. Fold induction of gene expression was calculated using the ΔΔCT method.

### Immunofluorescence (IF)

The immunofluorescence detection of IL-13, IL-4, IL-22, periostin, and IL-17 A was carried out on the lesional skin at W0 and W52. The nuclei were stained with DAPI (GIBCO, Grand Island, NY, USA) and observed by Leica DM2500 microscope (Leica Microsystems, Wetzlar, Germany).

### Enzyme-linked immunosorbent assay (ELISA)

Circulating protein levels of IL-13, IL-4, IL-22, IL-17 A and CXCL1 were assessed at W0 and W52 by ELISA (R&D Systems, Minneapolis, MN, USA), according to the manufacturer’s instructions.

### Statistical analysis

GraphPad Prism software (v.4.0; GraphPad Software Inc. La Jolla, CA, USA) was used for all statistical analyses. The Mann-Whitney test and Fisher test were used as appropriate to calculate statistical differences. A value of *p* < 0.05 was considered significant.

## Results

### Clinical response

A total of 50 elderly patients [34 males (68%)] had been treated with dupilumab during the reference period. The clinical characteristics of enrolled patients at baseline are reported in Table 1. In particular, 30 patients (60%) [19 (63.33%) males; age: 76.85 ± 6.13 years] had NF clinical phenotype (disease duration: 29.97 ± 15.21 months), and 20 (40%) [15 (75%) males; age: 74.44 ± 6.22 years] had F clinical phenotype (disease duration: 37.86 ± 18.22 months). 23 (46%) patients were aged between 65 and 74; 27 (54%) were 75 years or older. Most NF patients (20/30; 66.7%) had prurigo nodularis-like AD while the remaining had nummular eczema (5/30; 16.7%), head-and-neck dermatitis (2/30; 6.7%), hand dermatitis (2/30; 6.7%), or generalized eczema (1/30; 3.3%). The reported atopic comorbidities were rhinitis (19/50; 38%), asthma (11/50; 22%), and conjunctivitis (7/50; 14%). Other main comorbidities were hypertension (23/50; 46%), hypercholesterolemia (16/50; 32%), hypertriglyceridemia (10/50; 20%), diabetes mellitus (9/50; 18%), liver steatosis (6/50; 12%), benign prostatic hyperplasia (5/50; 10%), and chronic kidney failure (4/50; 8%). Regarding effectiveness parameters, mean EASI score at W0 was 25.12 ± 4.23 and significantly reduced to 8.26 ± 5.56 at W16 (*p* < 0.05) and 2.42 ± 3.15 at W52 (*p* < 0.05), respectively. P-NRS had a W0 mean value of 7.12 ± 2.64 versus 2.45 ± 1.78 at W16 and 1.03 ± 0.71 at W52 (*p* < 0.05). DLQI score at T0 was 22.84 ± 5.71 and significantly reduced to 7.15 ± 8.43 at W16 (*p* < 0.05), and 1.52 ± 0.93 at W52 (*p* < 0.05). No significant differences in the response to the treatment with dupilumab were found among the various phenotypes of AD. Regarding safety, registered AEs were conjunctivitis (7/50; 14%), worsening of head-and-neck dermatitis (3/50; 6%), and psoriasiform eruption (2/50; 4%), but none of them led to the discontinuation of dupilumab therapy.

**Table 1:** Clinical characteristics at baseline of AD elderly patients enrolled in the study

*N*	50		
**Male sex**,** number (%)**	34 (68%)		
**Age**, **years; mean +/- SD**	76.85 ± 6.13		
**Patients aged 65–74 years old; number (%)**	23 (46%)		
**Patients aged ≥ 75 years old; number (%)**	27 (54%)		
**Flexural clinical phenotype; number (%)**	20 (40%)		
**Non-flexural clinical phenotype; number (%)**	30 (60%)		
**Non-flexural clinical subtypes (30 patients)**			
Prurigo nodularis-like AD	20/30 (66.7%)		
Nummular eczema	5 (16.7%),		
Head-and-neck dermatitis	2 (6.7%),		
Hand dermatitis	2 (6.7%),		
Generalized eczema	1 (3.3%).		
**Atopic comorbidities number (%)**,			
Rhinitis	19 (38%)		
Asthma	11 (22%)		
Conjunctivitis	7 (14%)		
**Other comorbidities number (%)**			
Hypertension	23 (46%),		
Hypercholesterolemia	16 (32%)		
Hypertriglyceridemia	10 (20%)		
Diabetes mellitus	9 (18%)		
Liver steatosis	6 (12%)		
Benign prostatic hyperplasia	5 (10%)		
Chronic kidney failure	4 (8%)		
**Previous therapies number (%)**			
Topical corticosteroids	46 (92%)		
Topical calcineurin inhibitors	16 (32%)		
Cyclosporine	27 (54%)		
**Mean disease duration; mean (months) +/- SD**	31.87 ± 16.32		
**Baseline EASI score; mean +/- SD**	25.12 ± 4.23		
**Baseline P-NRS score; mean +/- SD**		7.12 ± 2.64	
**Baseline DLQI score; mean +/- SD**	22.84 ± 5.71		

AD: atopic dermatitis; EASI: Eczema Area and Severity Score; P-NRS: Pruritus-Numerical Rating Scale; DLQI: Dermatology Life Quality Index

### Dupilumab effects on cytokines modulation

**Fig. 1:**
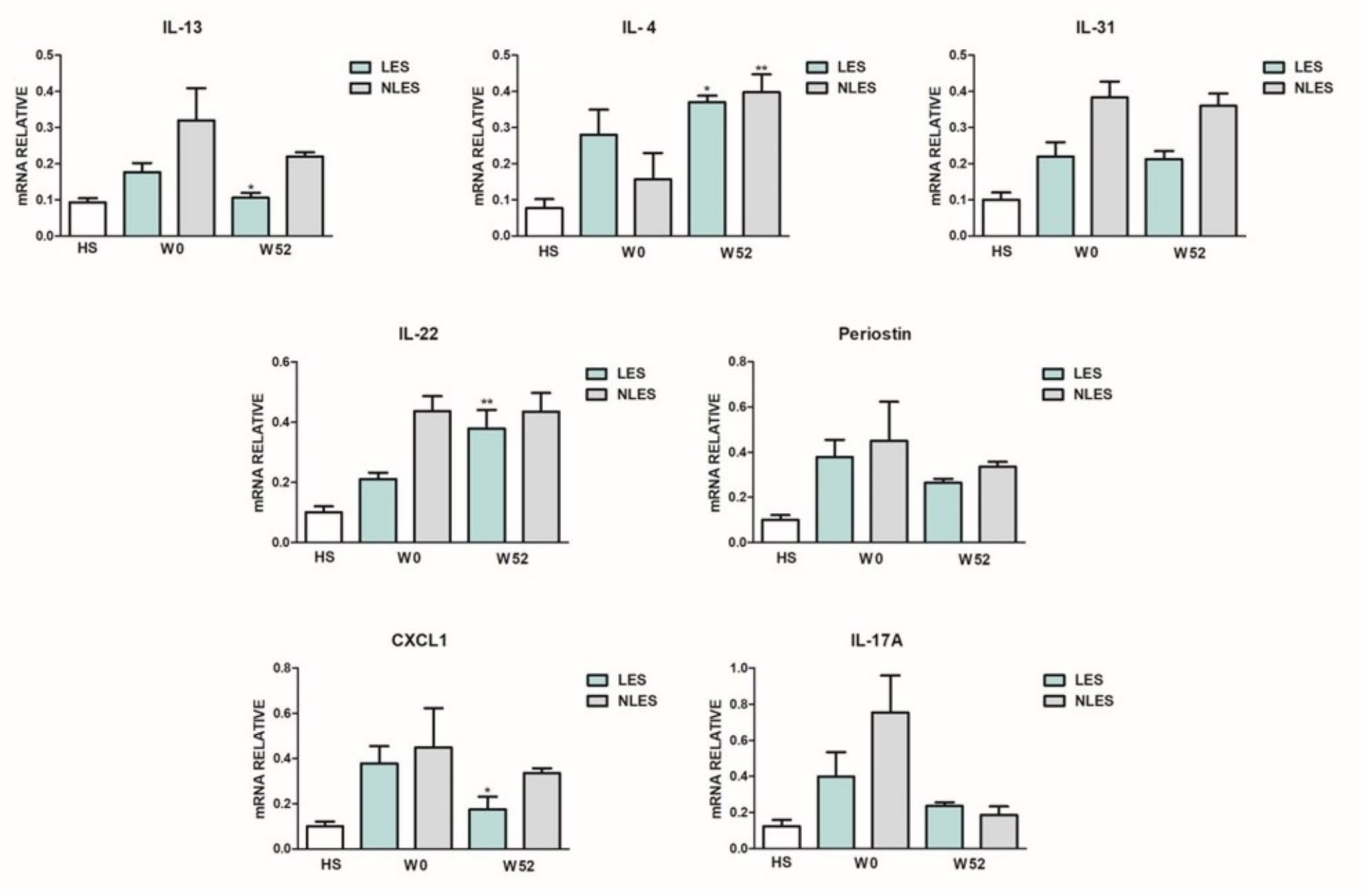
Gene expression profile of patients with phenotype non-flexural. Interleukin (IL)-13, IL-4, IL-31,IL-22, periostin, CXCL1 and IL-17 A, gene expression in skin of healthy subjects (HS), and in lesional (LES) and nonlesional (NLES) skin at baseline (W0) and after 52 weeks of dupilumab (W52). Values are normalized to the housekeeping gene 18 S and expressed as mean ± SD. Statistical significance was assessed using Mann–Whitney test

Gene expression of pro-inflammatory mediators in LES and NLES AD skin at W0 and W52 was evaluated. The aim was to relate the clinical effect of dupilumab with the different cytokine gene expression at W52 as compared to W0. The analysis was performed based on the clinical phenotype (NF or F). At W52, in NF group (Fig. 1) gene expression of IL-13, IL-31, periostin, CXCL1, and IL-17 A was downregulated, although not significantly, in LES as well as NLES skin, while that of IL-13 and CXCL1 only in LES skin (*p* < 0.05). Moreover, at W52 compared to W0 IL-4 gene expression was significantly up-regulated in LES (*p* < 0.05) as well as in NLES skin (*p* < 0.01), while IL-22 gene expression was significantly (*p* < 0.01) up-regulated in LES skin.

**Fig. 2:**
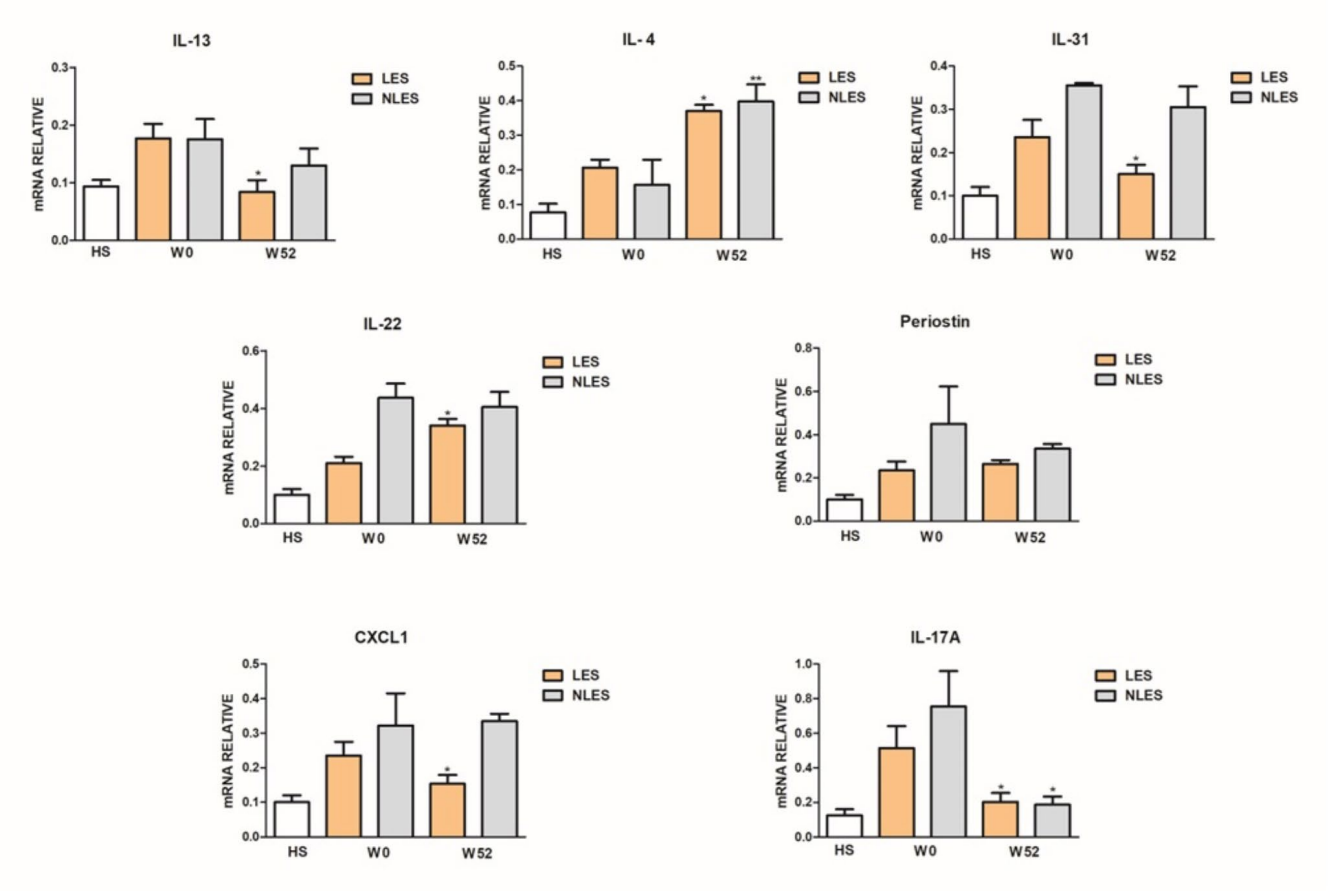
Gene expression profile of patients with phenotype flexural. Interleukin (IL)-13, IL-4, IL-31,IL-22, periostin, CXCL1 and IL-17 A, gene expression in skin of healthy subjects (HS), and in lesional (LES) and nonlesional (NLES) skin at baseline (W0) and after 52 weeks of dupilumab (W52). Values are normalized to the housekeeping gene 18 S and expressed as mean ± SD. Statistical significance was assessed using Mann–Whitney test

As regard clinical phenotype, at W52 respect to W0 the gene expression profile of patients of F group (Fig. 2) showed: not significant downregulation of IL-13, IL-31, periostin, CXCL1, and IL-17 A in both LES and NLES skin; significant (*p* < 0.05) decrease of IL-13, IL-31, and CXCL1 in LES skin; significant (*p* < 0.05) decrease of IL-17 A in LES as well as in NLES skin; significant (*p* < 0.05) upregulation of IL-4 in LES as well as in NLES; significant (*p* < 0.01) upregulation of IL-22 in LES skin.

**Fig. 3:**
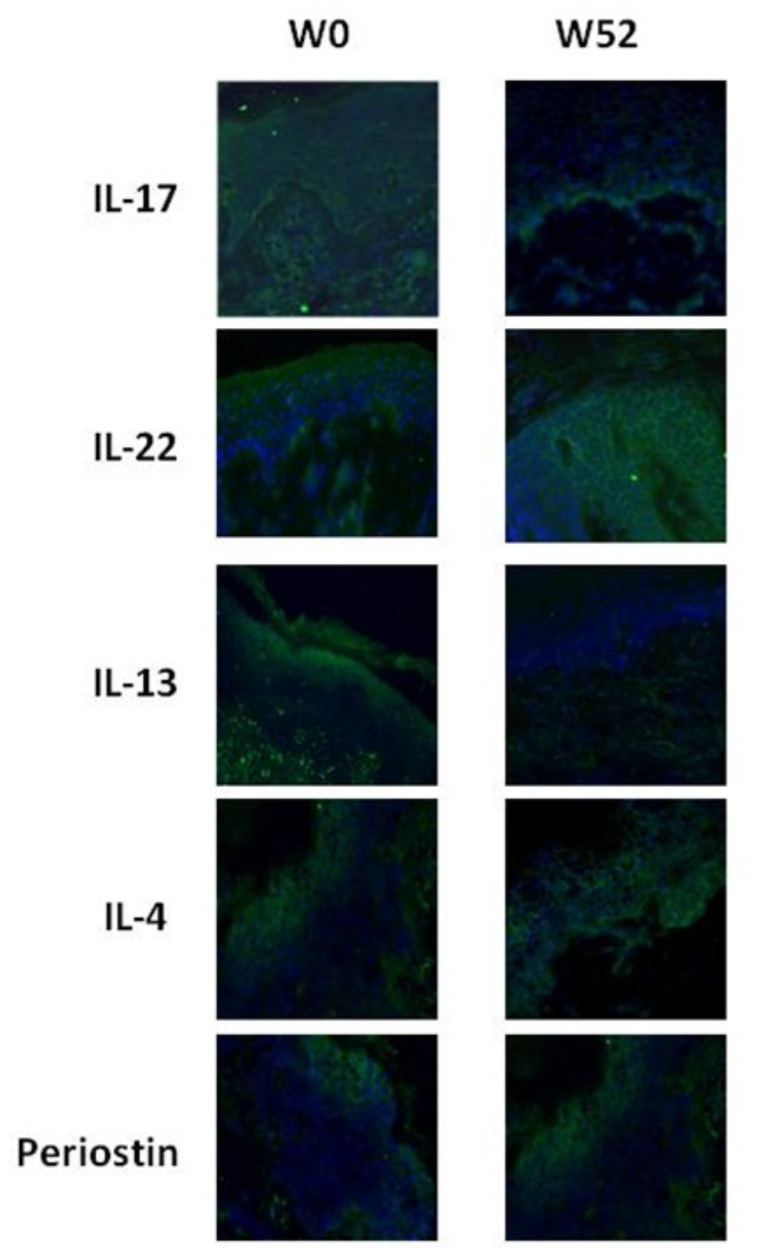
Immunofluorescence analysis in non flexural phenotype group of patients. IL-17, IL-22 IL-13, IL-4 and periostin cutaneous protein expression in lesional skin at W0 and W52 by immunofluorescence assay. Cell nuclei are counterstained with DAPI (blue). Magnification 20X

The immunofluorescence analysis was performed for both clinical phenotype (F group data not shown). In line with gene expression results, in the NF phenotype the protein levels of IL-13, IL-17 and periostin decreased at W52 compared to W0. As expected, an overexpression of both IL-4 and IL-22 after 52 weeks of therapy was found (Fig. 3).

**Fig. 4:**
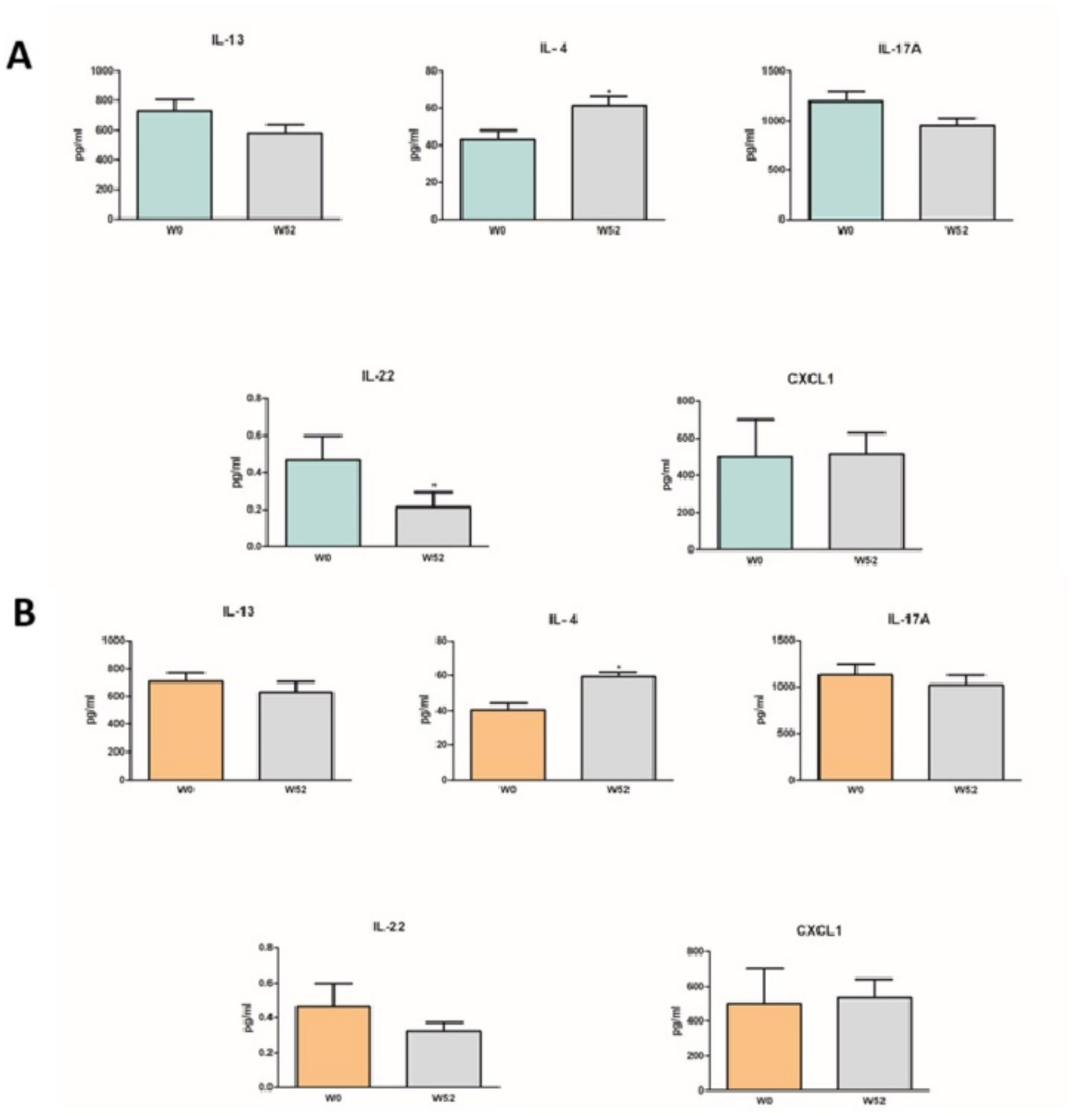
Protein levels of patients with e non-flexural (**a**) and flexural (**b**) phenotype. Interleukin (IL)-13, IL-4, IL-31, IL-17 A, IL-22 and CXCL1 circulating levels at W0 and W52 assessed by ELISA

Circulating levels of IL-13, IL-4, IL-17 A, IL-22, and CXCL1 were compared at W0 vs. W52. In the F group (Fig. 4a) as well as in NF group (Fig. 4b), we observed that levels of IL-17 A and IL-13 showed a decreasing trend, although not significant, at W52. In both groups IL-4 levels were significantly enhanced (*p* < 0.05) at W52. Moreover, IL-22 levels were significantly (*p* < 0.05) downregulated after 52 weeks of treatment in the F group, whereas IL-4 levels were not significantly decreased in the NF group. Regarding CXCL1 protein levels, dupilumab therapy seemed not to modulate the protein expression in both groups of patients.

## Discussion

The investigation into the long-term (52 weeks) effects of dupilumab-mediated IL4-Ra blockade on skin barrier impairment and immune response in elderly patients with F or NF AD is, in our opinion, an innovative approach. As previously reported, our data shows that dupilumab is an effective and safety treatment of AD also in elderly patients [13–18]. In fact, our study population achieved significant improvement in all clinical scores analyzed, with consistent and statistically significant reductions in EASI, P-NRS, and DLQI at each timepoint.

Previous studies illustrated that dupilumab selectively modulates Th2-, Th22-, and Th17-related inflammatory pathways in AD, as evidenced by transcriptome and proteomic analysis of both skin and blood at W4 and W16 [14].

Our findings demonstrate that after 52 weeks of dupilumab treatment, Th2 cytokines expression was decreased, with IL-13 and IL-31 downregulated in both patient groups at W52, at both gene and protein levels when compared with W0. Reduction in IL-13 has been associated with treatment response and improved clinical outcome [19]. It is noteworthy that previous evidence has shown a significant decrease in the expression of the itch-associated cytokine IL-31 as early as W16 in adult AD patients [14]. IL-31 contributes to itch and is noted to be elevated in the ageing population [8]. However, it remains to be clarified whether the intense itching is related to increased expression of IL-31 or of its receptors in elderly AD skin [8].

Our data also revealed a significant increase in both IL-4 gene expression and protein production at W52 when compared with W0. This finding is consistent with the existing literature, which demonstrates a significant increase in IL-4 levels as early as 4 weeks after dupilumab treatment initiation [20]. It is tempting to speculate that the observed increase in IL-4 under dupilumab might be explained by receptor-mediated accumulation, loss of feedback inhibition, and immune system compensation, rather than a sign of persistent Th2 inflammation. This might point to a functional inhibition of signaling rather than an increase in cytokine levels as part of the therapeutic response.

Dupilumab’s role in regulating the Th2 pathway is well established, and previous studies have also underscored its involvement in modulating the Th17/Th22 axis. Although the well-established role of IL-17 in AD, differences may be present in elderly patients due to factors such as immune-senescence (age-related changes in the immune system), alterations in skin barrier function associated with aging, comorbidities, and comorbidities systemic treatments. These factors may influence the expression and role of different mediators of inflammation such as IL-17 in this demographic population. Our results highlight a downregulation of IL17 expression at week 52 in the elderly population, further supporting trends observed in previously published studies in the adult population at both week 4 and 16 [14].

Unexpectedly, our results also revealed that IL-22 cutaneous expression was significantly increased, while circulating levels were decreased at W52 when compared to W0. In particular, lesional skin from NF group showed a more marked increase of IL-22 compared to F group. Conversely, while IL-22 circulating levels at W52 showed a significant decrease in NF patients when compared with W0, a reduction in IL-22 production was observed in F patients at the same time point. Given the IL-22 role in tissue remodelling and wound healing, it is tempting to speculate that IL-22 is recruited in situ to interact with fibroblasts to support this process. This speculation is further supported by the significant increase of IL-22 skin expression in NF AD with prurigo nodularis phenotype cohort when compared with the F AD, upon dupilumab treatment. Previous studies have emphasized the role of the Th22 subset in patients with prurigo nodularis, where IL-22 enhances keratinocyte proliferation while inhibiting terminal differentiation, leading to epidermal hyperplasia and acanthosis, hallmarks of prurigo nodularis lesions [21]. Additionally, IL-22 has been linked to wound healing and tissue remodelling by promoting epidermal regeneration in response to injury by stimulating keratinocyte migration and proliferation and by interacting with dermal fibroblasts, inducing extracellular matrix remodelling through upregulation of matrix metalloproteinases (MMPs) and collagen synthesis [22–24]. Therefore, the differential expression of IL-22 in NF versus F AD patients could indicate the presence of distinct remodelling processes [24–26]. Therefore, given the role of IL-22 in maintaining a delicate balance between epithelial regeneration and pathological hyperplasia, future studies with larger sample sizes and or multicenter collaborations would be needed to increase the statistical power to detect significant differences between NF and F phenotypes and draw definitive conclusions on the observed differences.

In conclusion, it is important to underline that, despite being in a demographic cohort characterised by inflammaging which entails an alteration in cytokines release, our results underscore the effectiveness of dupilumab at a late time point as W52. Of note, a dumping of the Th2- and Th17- immune response at both systemic and in situ level and a possible remodelling role of IL-22 in the skin is suggested.

## Data Availability

The datasets generated during and/or analysed during the current study are available from the corresponding author on reasonable request.
